# Psoriasis–Obesity‐Related Gene COX7C Promotes Keratinocyte Proliferation and Positively Regulates Inflammatory Responses in Psoriasis

**DOI:** 10.1111/jcmm.71277

**Published:** 2026-07-22

**Authors:** Fanghua Liu, Hang Su, Ruxue Han, Shougang Liu, Yongfeng Chen

**Affiliations:** ^1^ Department of Dermatology Guangdong Provincial People's Hospital Ganzhou Hospital (Ganzhou Municipal Hospital) Ganzhou Jiangxi Province People's Republic of China; ^2^ Department of Dermatology The People's Hospital of Longhua Shenzhen Guangdong Province People's Republic of China; ^3^ Department of Dermatology Air Force Medical Center, PLA Beijing People's Republic of China; ^4^ Department of Dermatology Dermatology Hospital of Southern Medical University Guangzhou Guangdong Province People's Republic of China

**Keywords:** COX7C, obesity, psoriasis, ScRNA‐seq analysis, transcription factors

## Abstract

Obesity (OB) predisposed to psoriasis (PSO) and aggravation of existing PSO. It is a risk factor for PSO and metabolic syndrome. However, the biological link between PSO and OB remains to be studied. The study aimed to explore the relationship between PSO and OB, identify potential predictive biomarkers, and investigate their expression and function in PSO. Mendelian randomization (MR) was used to observe the causal effect between OB and PSO. Using a public database, we identified key shared genes in PSO and OB. Functional enrichment analysis of key shared genes was performed. Single‐cell RNA sequencing (ScRNA‐seq) analysis was used to further analyse the pivotal biomarkers at the skin cell level in PSO. Motif enrichment analysis was used to screen key transcription factors (TFs). The functional characteristics of PSO skin cell subtypes were evaluated from a metabolic perspective. Two machine learning algorithms were used to screen diagnostic characteristic biomarkers for PSO‐OB. The diagnostic efficacy of biomarkers was evaluated by logistic regression analysis and subject's working characteristic curve. Construct a cell model of PSO, and screen the significantly upregulated or downregulated target genes. Knock down the overexpressed target genes using siRNA to investigate their effects on the proliferation and inflammatory response of PSO cells, and clarify their role in the pathogenesis of PSO. MR Analysis showed that OB was a high‐risk factor for PSO vulgaris. Nineteen key differentially expressed genes (DEGs) were screened between PSO and OB. Functional enrichment analysis showed that these key DEGs were significantly related to lipid metabolism, thermogenesis and ras signalling pathways. According to ScRNA‐seq analysis, psoriatic skin cells were mainly composed of keratinocytes (KC), dendritic cells (DC), macrophages, natural killer cells (NK‐cells), T cells, Treg cells and melanoma. Among them, the key DEG of PSO‐OB COX7C was significantly correlated and enriched in PSO skin cell development. We identified 24 active transcriptional regulators behind the cellular diversity of psoriatic skin subtypes. We evaluated the role of psoriatic skin cell subtypes in terpenoid backbone biosynthesis, steroid hormone biosynthesis, propanoate metabolism and N‐glycan from the perspective of metabolism abundant functions in lipid metabolism such as biosynthesis. Six key genes (AATF, COX7C, GHRH, KCNA3, PCSK1 and PLIN1) obtained by least absolute shrinkage and selection operator (LASSO) regression and support vector machine recursive feature elimination (SVM‐RFE) algorithm can be used as promising diagnostic biomarkers for PSO and OB. COX7C significantly upregulates and promotes the proliferation of psoriatic cells while also positively regulating the inflammatory response in PSO. Overall, our study provides a comprehensive assessment of the relationship of potentially key genes in PSO and OB to lipid metabolism, psoriatic skin subtype cells and TFs. COX7C is a diagnostic biomarker for PSO‐OB and positively regulates the proliferation and inflammatory response of PSO cells. Which may pave the way for exploring possible associations in the development of these two diseases.

## 1 Introduction

PSO is a common systemic chronic, recurrent and inflammatory disease in dermatology. Its causes are complex, involving genetic predisposition, environmental triggers and immune disorders. PSO affects 2%–4% of the general population [1]. It not only affects the health of the skin but also may involve other systems or organs during the course of the disease. It leads to a range of related diseases such as OB, cardiovascular disease, metabolic syndrome, psoriatic arthritis, depression and anxiety. Severe PSO severely affects patients' quality of life and social activities.

In recent decades, the prevalence of immune‐mediated inflammatory diseases has continued to increase [2], and research evidence suggests that the epidemiology of PSO is changing. According to the Global Burden of Disease Study 2019 (GBD 2019), the prevalence of PSO in China is estimated to be 0.56%, with more than 4.6 million cases of PSO worldwide [3]. Given that the genetic basis remains unchanged, there is a growing awareness that environmental factors, including lifestyle, play an important role in this growing prevalence [4]. In industrialized countries, diets high in fat, salt and sugar, as well as excessive calorie intake and the inverse ratio of food intake to energy expenditure, have led to the OB epidemic [5]. Epidemiological studies have shown that compared with individuals without PSO, patients with PSO may be more obese and have a greater risk of such metabolic changes [6].

Studies have shown that TNF‐α and IL‐6 secreted by adipose tissue may lead to the inflammatory state of PSO, and both leptin and resistin have high levels in obese patients with PSO, and their plasma concentrations are correlated with the severity of PSO [7]. OB is not only closely related to the incidence and severity of PSO, but also affects the response to treatment. Studies have shown that the proportion of patients with a 75% reduction in PSO area and severity index decreases with the increase in body mass index (BMI) [8, 9]. Obese patients with PSO are less effective with conventional systemic drug treatment, with a higher risk of adverse reactions and increased treatment costs [10]. Low‐grade chronic inflammation is the common pathophysiological mechanism of OB and PSO, and OB‐related inflammatory states are considered to be the link between PSO and metabolic syndrome [11, 12].

MR uses genetic variants closely associated with exposure factors as instrumental variables [13], which can effectively reduce the influence of confounding factors on causal inference and is widely used to verify causal relationships in observational studies [14]. In this study, the two‐sample MR method was applied to investigate the causal association between OB and PSO and its subtypes, providing genetic evidence for clarifying the pathogenesis and clinical intervention of these diseases.

Integrated bioinformatics analysis is a technique based on gene sequencing data to discover disease‐related genes and explore possible mechanisms. ScRNA‐seq is gene sequencing performed on a single‐cell basis, where differences can be found at the cellular level. In this study, a public database was used to identify common DEGs in PSO and OB, and the main biological functions regulated by DEGs were analysed for enrichment analysis. At the same time, further analysis using the scRNA‐seq dataset revealed the developmental trajectory of various cell subpopulations in psoriatic skin and identified their key transcriptional regulatory targets to explore possible associations in the development of the two diseases.

The IL‐23/IL‐17 axis is the main pathogenic pathway of PSO, with IL‐23 and IL‐17A playing a central role. Inflammatory cytokines TNF‐α, IL‐1β and IL‐6 synergistically enhance the inflammatory response, regulate excessive proliferation of keratinocytes, and participate in immune dysregulation. These cytokines are closely related to the pathogenesis of PSO and are potential targets for targeted therapy. This study focuses on screening out biomarker target genes with significant expression differences related to OB and PSO, and through in vitro cell experiments, explores the regulatory effects of key target genes on the proliferation and differentiation of keratinocytes and the inflammatory response of PSO, clarifying their functions and regulatory mechanisms.

## 2 Methods

### 2.1 MR Analysis

#### 2.1.1 Research Design

The research of MR is mainly based on three main assumptions: (1) IV is correlated with exposure factors; (2) independence hypothesis: IV directly affects the outcome only through exposure; (3) exclusivity hypothesis: IV is not related to potential confounders of outcomes. In this study, we used OB as exposure, collected PSO (including some subtypes) as outcome, performed a comprehensive MR Analysis, performed a sensitivity analysis, and performed a reverse MR Analysis. The research design framework is shown in Figure S1.

#### 2.1.2 Date

In this study, PSO‐related diseases: PSO (total), guttate PSO, PSO vulgaris and PSO vulgaris (strictly defined) were derived from the Finnish database (R11) (Table S1). OB data are derived from the ‘LeeLab’ genome‐wide association study (GWAS) aggregated statistics (Table S1). All GWAS were performed in European populations. Each GWAS has been approved by its respective ethics committee.

#### 2.1.3 Choice of Instrumental Variable (IV)

First, we selected SNPs that were significantly associated with OB and PSO‐related diseases (GWAS) (*p* < 5 × 10^−6^). After excluding SNPs with linkage disequilibrium (LD), the conditions for linkage disequilibrium (LD) of the selected SNPs should satisfy *r* ^2^ < 0.01 and distance > 10,000 kb. Second, we calculated the interpreted variance (*R* ^2^) and *F* statistical parameters to determine that the identified IV is strongly associated with exposure (Table S2). In general, SNPs with an *F*‐statistical parameter > 10 are considered strong tools.

#### 2.1.4 Analytical Method

To estimate the causal effects of OB and PSO‐related diseases, we performed a two‐sample two‐way MR Analysis. The causal effect was estimated by five efficient methods: the multiplicative random effects model of inverse variance weighting (IVW) method, the weighted median method, the weighted mode, the MR‐Egger regression, and the MR Multidirectional residual sum and outlier (MR‐PreSO) method. In addition, Cochran's *Q* test was used in the IVW to examine heterogeneity among the SNPs included in each analysis. The intercept of the MR‐Egger method is used to evaluate the tool strength independent direct effects (InSIDE) hypothesis, which assumes horizontal multidirectional effects independent of variant‐exposure associations. The MR‐PreSO global test is also used to evaluate global horizontal multidirectivity. At the same time, MR‐PreSO corrected for horizontal pleiotropy by removing outliers. Among them, inverse variance weighting (IVW) is the basic analysis method. When the *p*‐value of IVW is less than 0.05, and the direction of IVW and MR‐Egger is consistent, the result is statistically significant. All analyses were performed using R (v4.3.3) statistical software. The MR Analysis was performed using the R‐based software package ‘TwoSampleMR’. The ‘MR‐PreSO’ package is used for multiplicity testing.

### 2.2 Microarray Data Resources and Processing

Microarray datasets were downloaded from the Gene Expression Omnibus (GEO) database (accession codes: GSE13355, GSE447751, GSE50790 and GSE30999) and have been processed by Robust Multi‐array Average (RMA) normalization (Table 1). In each data set, only PSO lesion samples and normal skin tissues were selected. We combined the GSE13355, GSE447751 and GSE50790 microarray datasets as the test set and the GSE30999 microarray dataset as the validation set.

**TABLE 1:** GEO database microarray data set queue characteristics.

Data set	Platform	Platform name	Sample
GSE13355	GPL570	[HG‐U133_Plus_2] Affymetrix Human Genome U133 Plus 2.0 Array	PSO (*n* = 58), normal (*n* = 64)
GSE47751	GPL570	[HG‐U133_Plus_2] Affymetrix Human Genome U133 Plus 2.0 Array	PSO (*n* = 6), normal (*n* = 6)
GSE50790	GPL570	[HG‐U133_Plus_2] Affymetrix Human Genome U133 Plus 2.0 Array	PSO (*n* = 4), normal (*n* = 4)
GSE30999	GPL570	[HG‐U133_Plus_2] Affymetrix Human Genome U133 Plus 2.0 Array	PSO (*n* = 85), normal (*n* = 85)

### 2.3 Date Quality and DEGs

Using the online tool Sangerbox (a free online platform for data analysis, http://vip.sangerbox.com/), hierarchical cluster analysis (HCA) and principal component analysis (PCA) were performed on the test set dataset to detect data quality. Linear models for microarray data (Limma) is a differential expression screening method based on generalized linear models. DEGs were performed using the IMMA rapid differential analysis tool in the Sangerbox. The data were screened for DEGs between the PSO skin and normal skin with criteria of fold change (FC) > 2 and *p*‐value < 0.05.

### 2.4 Weighted Gene Co‐Expression Network Analysis (WGCNA)

WGCNA can cluster genes with similar expression patterns and analyse associations between modules and specific traits or phenotypes. Use the ImageGP tool: An easy‐to‐use data visualization web server for scientific researchers. iMeta 1: e5. The Pearson coexpression correlation matrix was constructed for all the paired genes in the PSO test set, converted into an adjacency matrix, and then converted into a topological matrix to identify the module genes significantly associated with the clinical features of PSO.

### 2.5 PSO‐OB DEGs

OB‐related genes from GWAS directory website (https://www.ebi.ac.uk/gwas/) to download. DEGs were intersected with key module genes and OB‐related genes to screen PSO‐OB DEGs.

### 2.6 Expression Level and Diagnostic Value

The receiver operating characteristic curve (ROC) was plotted by using the ‘pROC’ package in R software in the test set of PSO. The higher the area under the curve (AUC) indicates increasing diagnostic values. And validate the expression levels of DEGs in psoriatic lesions and normal skin tissues in the validation set.

### 2.7 Functional and Pathway Enrichment Analysis via GO, KEGG, GSVA and GSEA

The Gene Ontology (GO) (http://geneontology.org/), Kyoto Encyclopedia of Genes and Genomes (KEGG) (https://www.kegg.jp/), Gene Set Variation Analysis (GSVA) and Gene set enrichment analysis (GSEA) were performed using the R ‘clusterProfiler’ (https://bioconductor.org/) package to investigate the functional roles and pathway signalling relevance of the DEGs. The ‘DOSE’, ‘ggplot2’, ‘pathview’, ‘org.Hs.eg.db’, ‘enrichplot’, ‘pathwork’, ‘GSVABase’ and ‘GSEABase’ packages of R software were used to visualize the plots. *p* < 0.05 was considered statistically significant.

### 2.8 scRNA‐Seq Data Analysis and Cell Type Annotation

To fully understand psoriatic skin cells, we from a GEO database (https://www.ncbi.nlm.nih.gov/geo/query/acc.cgi) to download GSE151177 data set (13 cases of human PSO skin and 5 cases of healthy volunteers) as a single‐cell sequencing data set. Using the Seurat (v.4.3.0) package in R software for analysing the RNA‐SEQ data, Seurat objects are generated on RStudio (v.4.2) as input files. Normalization, feature selection of highly variable genes, Uniform Manifold Approximation and Projection (UMAP) dimensionality reduction clustering, and feature gene identification were performed on the data of scRNA‐seq. The ‘FindAllMarkers, FindMarkers’ function and ‘COSG algorithm’ were used in the Seurat package to identify marker genes for different cell clusters and cluster DEGs for all cell clusters.

### 2.9 CytoTRACE Analysis

On the scRNA‐seq data, Monocle and CytoTRACE in R software were used to construct cell development trajectory and reconstruction analysis, and the cells with the greatest differentiation potential were identified. Consider the number of genes expressed by each cell as a determinant of its developmental potential.

### 2.10 Analysis of Transcriptional Gene Regulatory Network

The TFs of each cell were reconstructed using the pySCENIC (v0.10.3) algorithm for the scRNA‐seq data; the differences in regulatory activity between cell groups were compared using the empirical Bayesian contraction method of the R package ‘limma’, and the difference values between cells from one cluster and cells from all other clusters were calculated.

### 2.11 Gene Signature Screening and Verification

Two machine learning algorithms, including LASSO logistic regression and SVM‐RFE, were independently used to screen diagnostic genes from intersections. The ‘glmnet’ package, ‘e1071’ and ‘msvmRFE’ packages in the R software package are used for the two calculation methods, respectively. Lambda values with minimum classification error are found to identify the gene selection that overlaps between the two algorithms for variables as diagnostic biomarkers. The predictive utility of genes was estimated using ROC curve analysis, and AUC values were computed using the ‘pROC’ package. The ‘rms’ package was used to construct a nomogram based on the expression patterns of shared diagnostic genes. The area under the ROC curve was calculated to evaluate the diagnostic efficiency of the nomogram.

### 2.12 Molecular Docking

Ten unsaturated fatty acids with potential relevance to PSO were identified from the STICH database using predefined selection criteria, including literature‐based association with PSO pathogenesis, experimental evidence and biological relevance. The 10 unsaturated fatty acids included: Arachidonic acid, linoleic acid, Alpha‐Linolenic acid, Eicosapentaenoic acid, Docosahexaenoic acid, Gamma‐Linolenic acid, Oleic acid, Palmitoleic acid, Conjugated Linoleic acid and Stearidonic acid. Autodock vin was used for batch docking, and the unsaturated fatty acids with the best docking affinity for each gene were screened, and then their Autodocktools docking was performed one‐to‐one.

### 2.13 Cell Culture and Treatment

Human immortalized keratinocytes (HaCaT) were cultured in high‐glucose Dulbecco's Modified Eagle Medium (DMEM) supplemented with 10% fetal bovine serum (FBS) and 1% penicillin–streptomycin. The cells were maintained in a humidified incubator at 37°C with 5% CO_2_. Cells in the logarithmic growth phase with viability ≥ 95% were used for subsequent experiments. To induce PSO‐like characteristics in vitro, HaCaT cells were stimulated with 100 ng/mL IL‐22 (tzybiotech) or 100 ng/mL lipopolysaccharide (LPS; Biosharp) for 12 or 24 h. The blank control group was treated with an equal volume of the vehicle.

### 2.14 RNA Extraction and Quantitative Real‐Time PCR (qRT‐PCR)

Total RNA was extracted from the treated HaCaT cells using the Trizol reagent according to the manufacturer's protocol. The extracted RNA was subsequently reverse‐transcribed into complementary DNA (cDNA). Quantitative real‐time PCR (qRT‐PCR) was performed utilizing the SYBR Green method to detect the mRNA expression levels of candidate genes (*AATF*, *COX7C*, *GHRH*, *KCNA3*, *PCSK1* and *PLIN1*) and inflammatory factors (*IL‐17A*, *IL‐23*, *IL‐1β*, *TNF‐α* and *IL‐6*). GAPDH was employed as the internal reference gene. The relative mRNA expression levels were calculated using the $2^{\left(-∆∆C_{t}\right)}$ method. The specific primer sequences used in this study are detailed in Table 2.

**TABLE 2:** Primer sequences used for qRT‐PCR.

Gene name	Forward primer (5′ → 3′)	Reverse primer (5′ → 3′)
COX7C	AGGAGCCACTATGAGGAGGG	GGTGTAGCAAATGCAGATCCA
GHRH	AGAGAGCAACCAAGAGCGAG	CATCCCTGGGAGTTCCTGTG
AATF	AGATCCTTCCTCAAGCCCCT	GTTCTGTCCGAAGTTCTCTGC
KCNA3	GGGATCTCTCTGTGCCATCG	CCACGTGCATGTACTGGGAT
PCSK1	GGGGAAGGGATCTATTTCTTTCTTG	AAGGCACTCCTTCGAGACCT
PLIN1	TGTCTCCTCAACCAAGGGGA	ATGTCCCGGAATTCGCTCTC
IL‐6	ACTCCTTCTCCACAATACCCC	GATGCCGTCGAGGATGTACC
IL‐1β	CCCTCTGTCATTCGCTCCC	TAAAGAGAGCACACCAGTCCA
IL‐23	CCAGCTTCATGCCTCCCTAC	CTGGAGGGTGCGAAGGATTT
IL‐17A	TCCCACGAAATCCAGGATGC	GCACTTTGCCTCCCAGATCA
TNF‐α	GCCCATGTTGTAGCAAACCC	GGAGGTTGACCTTGGTCTGG
GAPDH	GGAGTCCACTGGCGTCTTCA	GTCATGAGTCCTTCCACGATACC

### 2.15 siRNA Transfection

HaCaT cells were seeded into culture plates and grown to 50%–60% confluence. The cells were randomly divided into three groups: a blank control group, a negative control siRNA (siRNA‐NC) group, and a *COX7C*‐specific siRNA (siRNA‐*COX7C*) group. Transfection was performed using liposomes strictly according to the manufacturer's instructions. At 48 h post‐transfection, the cells were harvested, and the knockdown efficiency was verified via qRT‐PCR. A reduction in relative target mRNA expression of ≥ 50% combined with a statistically significant difference (*p* < 0.05) was considered a successful knockdown for subsequent functional assays.

### 2.16 Functional Experiments (Cell Proliferation and Inflammatory Assays)

To investigate the role of the target genes in HaCaT cells exhibiting PSO‐like characteristics, functional experiments including cell proliferation and inflammatory factor secretion were performed comprehensively.

#### 2.16.1 Cell Proliferation Assays

HaCaT cells (blank control, IL‐22, siRNA‐NC + IL‐22 and siRNA‐*COX7C* + IL‐22 groups) were seeded in triplicate. After 12 h of stimulation with IL‐22 (100 ng/mL), cell viability was assessed using an enhanced Cell Counting Kit‐8 (CCK‐8; Biosharp). Additionally, cell proliferation was visualized and quantified using the Click‐iT EdU‐594 Cell Proliferation Imaging Kit (Biosharp). The EdU‐positive rate was calculated to evaluate the proliferative capacity.

#### 2.16.2 Inflammatory Factor Detection

Seven groups of cells (including relevant controls, IL‐22 stimulated and LPS stimulated groups with or without siRNA transfection) were set up in triplicate. After 12 h of the respective stimulations, cell culture supernatants were collected. The concentrations of secreted inflammatory cytokines including IL‐17 (CHE0054), IL‐1β (CHE0001), IL‐6 (CHE0009), IL‐23 (CHE0008) and TNF‐α (CHE0019), were measured using specific Enzyme‐Linked Immunosorbent Assay (ELISA) kits (abio) according to the manufacturer's guidelines. Concurrently, the intracellular mRNA expression levels of these inflammatory factors were quantified via qRT‐PCR as described above.

### 2.17 Statistical Analysis

All experiments were performed with at least three technical replicates (three replicate wells). Data were analysed using SPSS software. The results were presented as mean ± standard deviation (SD). Differences between groups were analysed using appropriate statistical methods (e.g., Student's *t*‐test or one‐way ANOVA). A value of *p* < 0.05 was considered to indicate a statistically significant difference.

## 3 Results

### 3.1 OB Is a High‐Risk Factor for PSO Vulgaris

**FIGURE 1:**
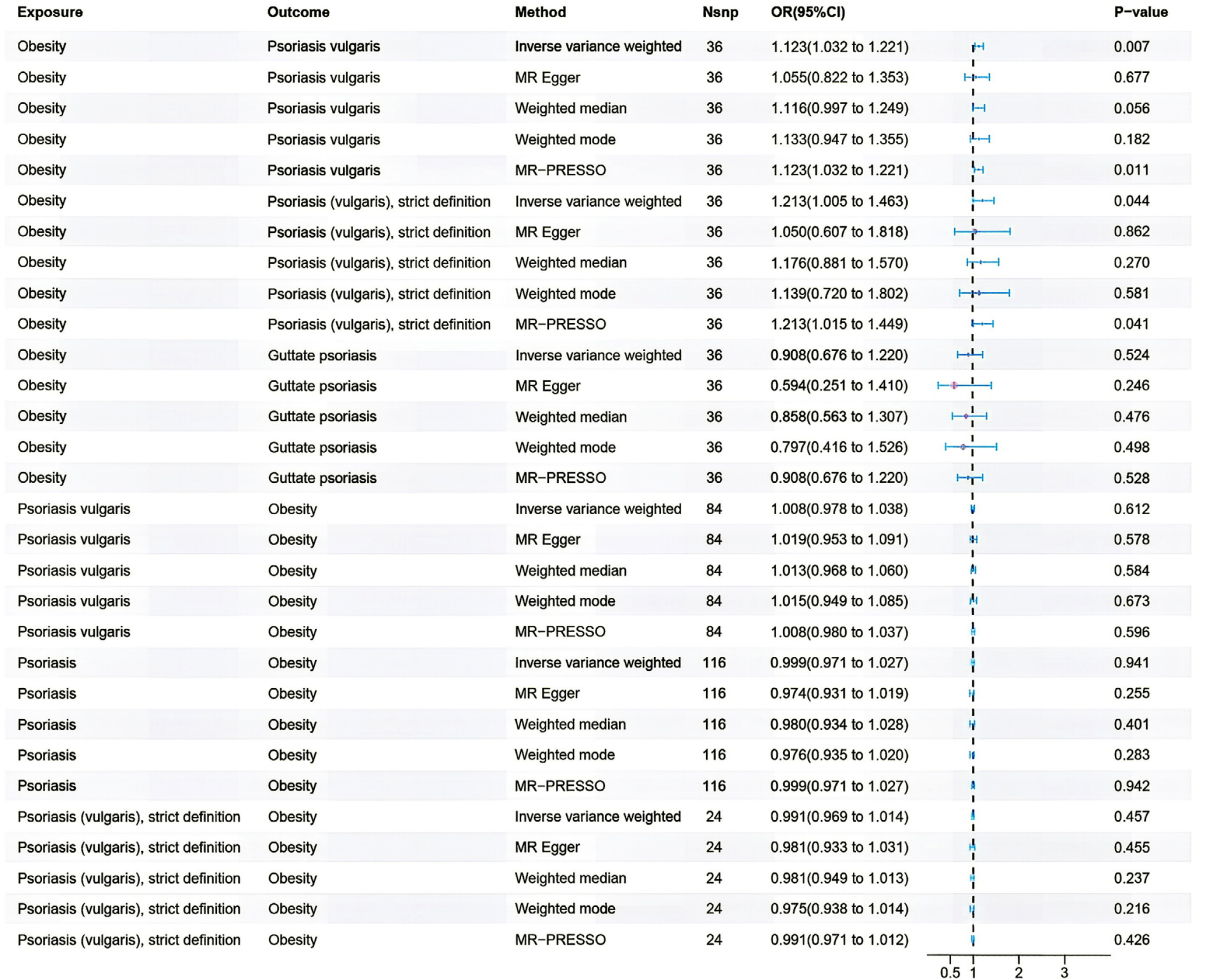
Forest map of causal effects of OB‐induced PSO (including reverse MR Analysis). The red circles represent the risk estimates for each study; the Horizontal line 95% confidence interval. OR, odds ratio.

We performed a two‐way MR Analysis of OB and PSO‐related diseases (Figure 1). In positive MR Analyses of OB and PSO (total), guttate PSO, PSO vulgaris and PSO vulgaris (strict definition), We found that OB (OR = 1.123, 95% CI = 1.032–1.221, *p*‐value = 0.007) was a high‐risk factor for the development of PSO vulgaris. At the same time, OB (OR = 1.213, 95% CI = 1.005–1.463, *p*‐value = 0.044) is also a high‐risk factor for PSO (Vulgaris), strict definition. There is no causal relationship between OB and guttate PSO. OB was removed in the opposite direction to the β value of PSO (overall). In reverse MR Analysis, we did not find a causal relationship between PSO‐related diseases and OB. Guttate PSO has the opposite beta value of OB and is removed. Sensitivity analysis also supported the above results (Table S3).

### 3.2 The 19 PSO‐OB Key Genes Have Diagnostic Specificity for PSO

**FIGURE 2:**
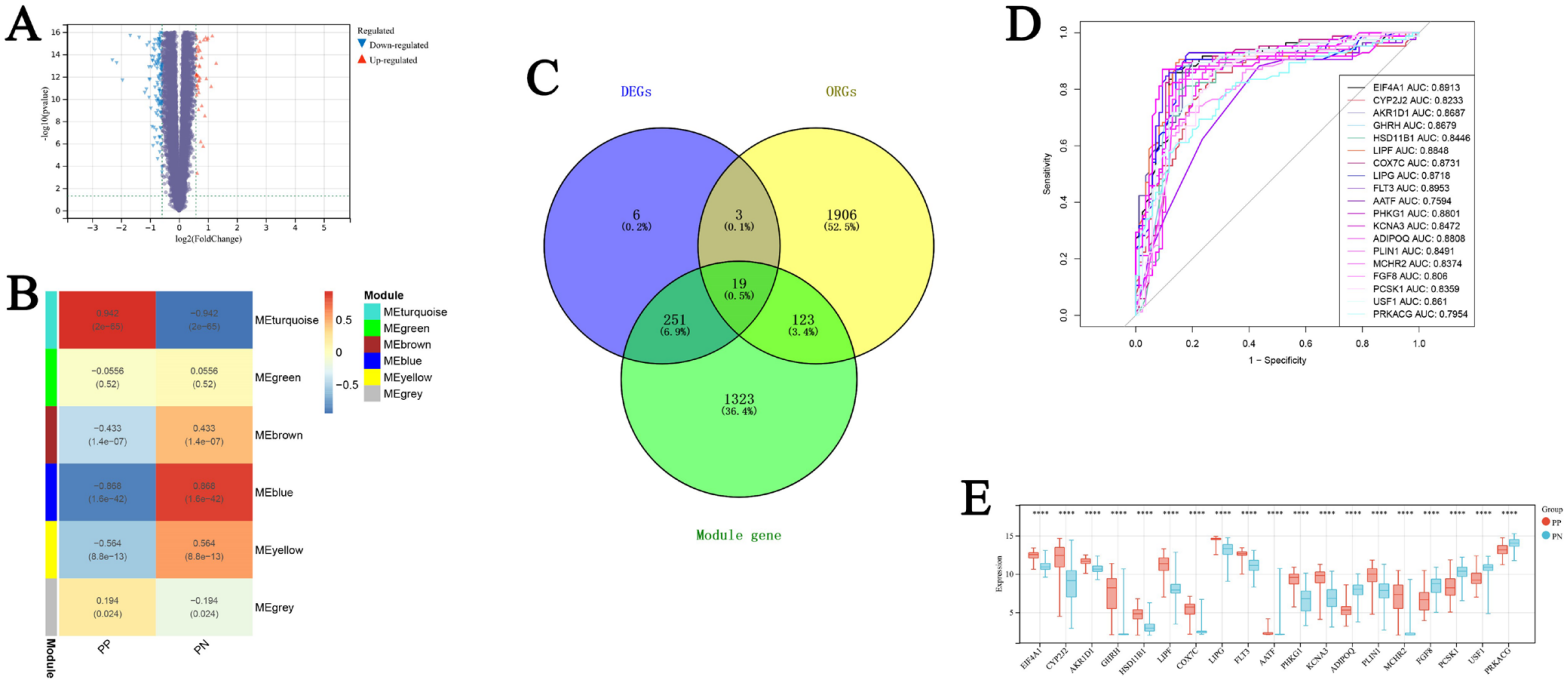
Screening and verification of key DEGs of PSO‐OB. (A) Distribution of DEGs in the volcano map PSO trial dataset. (B) Heat map shows the correlation between the WGCNA module and PSO. (C) Venn diagram shows the intersection genes of PSO‐DEGs, PSO key module genes and OB‐related genes. (D, E) PSO‐OB key DEGs, ROC curve, and expression level bar chart.

In the PSO microarray test set, 279 DEGs were identified by comparing psoriatic patients' lesions (PP) and normal skin tissues (PN) (|log_2_(fold change)| > 1, adjusted *p* < 0.05) and visualized via a volcano plot (Figure 2A). WGCNA clustered genes with similar expression patterns into 6 PSO‐related co‐expression modules, among which 1716 hub genes in the blue and green core modules were candidate genes closely related to PSO pathogenesis (Figure 2B). By intersecting three gene sets—279 DEGs, 1716 hub genes from WGCNA core modules, and 2051 ORGs—19 specific PSO‐OB key genes (EIF4A1, CYP2J2, AKR1D1, GHRH, HSD11B1, LIPF, COX7C, LIPG, FLT3, AATF, PHKG1, KCNA3, ADIPOQ, PLIN1, MCHR2, FGF8, PCSK1, USF1 and PRKACG) were identified (Figure 2C). ROC and differential expression analyses using PSO validation dataset (GSE30999) showed all genes had AUC > 0.7 and significant differential expression (***p < 0.001), confirming their stability and specificity (Figure 2D,E).

### 3.3 PSO and 19 Key PSO‐OB Genes Are Closely Related to the Abnormal Activation of Lipid Metabolism Pathways

**FIGURE 3:**
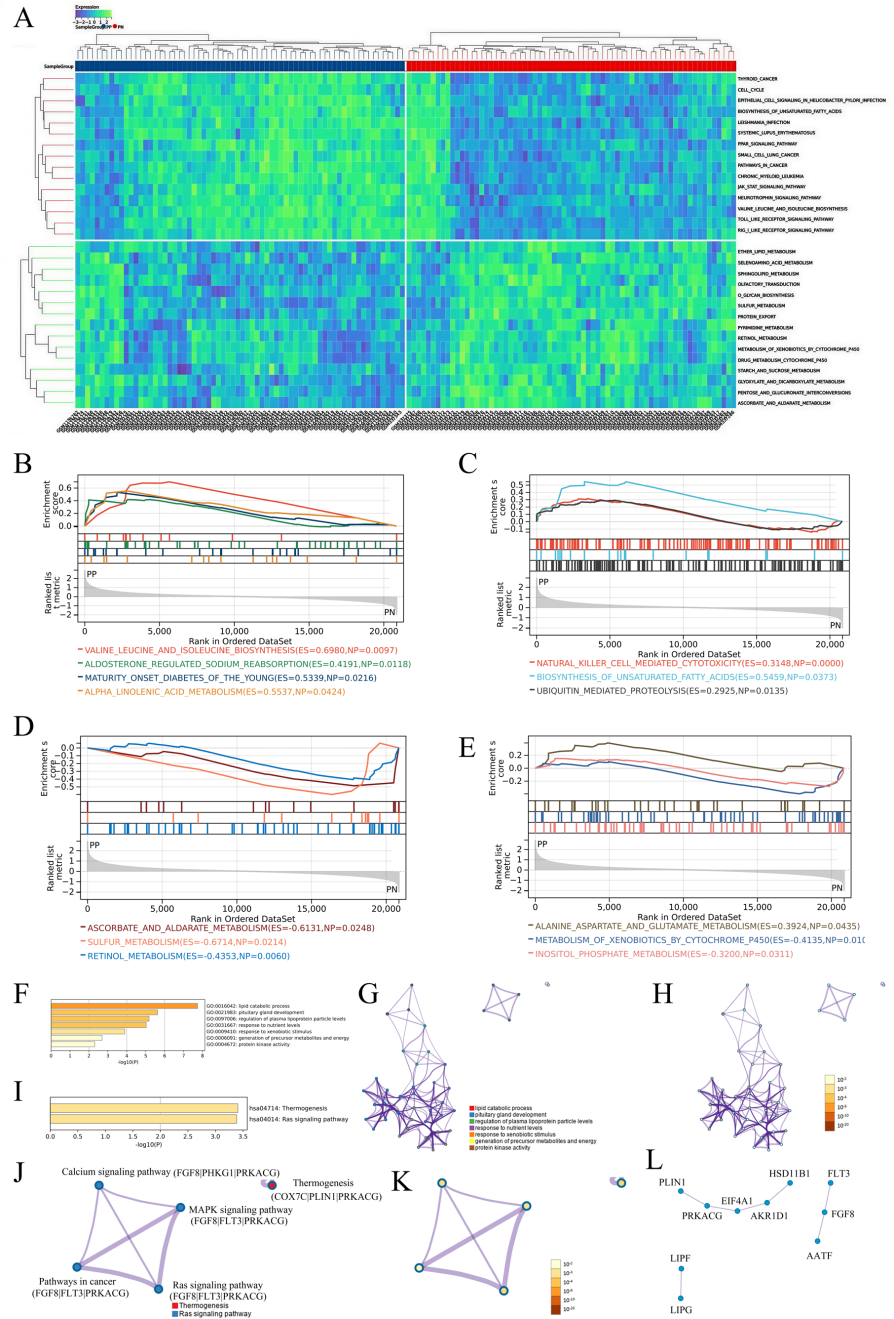
Functional enrichment analysis of lipid metabolism pathways and 19 key genes in PSO. (A) GSVA analysis of potential biological pathways in samples of different clusters of PSO. (B–E) The differences in lipid metabolism‐related signalling pathways between PP and PN of GSEA analysis. Enrichment score (ES) and normalized *p*‐value (NP), psoriatic patients (PP) and psoriatic non‐lesional skin (PN)are indicated. (F–H) GO functional enrichment analysis of the selected 19 key genes. (I–K) KEGG functional enrichment analysis of the 19 key genes. (L) PPI relationships among the 19 key genes analysed by STRING.

To explore the potential biological mechanism of PSO, we conducted GSVA based on the gene expression profiles of different clusters in the PSO test set, aiming to identify the biological pathways that are abnormally regulated in PSO. The results showed that the lipid metabolism‐related signalling pathways associated with the progression of PSO were significantly correlated (Figure 3A). To further confirm the differences in lipid metabolism pathways among different groups, we conducted GSEA. The results of GSEA showed that multiple pathways related to lipid metabolism were significantly enriched in the PSO samples. Including aldosterone regulated sodium reabsorption, valine leucine and isoleucine biosynthesis, alpha linolenic acid metabolism, maturity on diabetes of lung, biosynthesis of unsaturated fatty acids, ubiquitin mediated proteolysis, natural killer cell mediated cytotoxicity, ascorbate and aldarate metabolism, retinol metabolism, sulphur metabolism, alanine mediated metabolysis aspartate and glutamate metabolism, metabolism of xenobiotics by cytochrome p450, inositol phosphate metabolism (Figure 3B–E).

We also performed functional enrichment analysis on the selected 19 key genes. GO analysis showed that 19 key genes were associated with the biological processes lipid catabolic process, pituitary gland development, regulation of plasma lipoprotein particle levels, response to stimulus levels, response to xenobiotic, generation of precursor nutrients and metabolites, and protein kinase activity, indicating that these key genes were involved in lipid metabolism (Figure 3F–H). In addition, KEGG analysis showed that these 19 key genes were mainly associated with thermogenesis and Ras pathway signalling (Figure 3I–K). The PPI relationships among 19 key genes were analysed by STRING, and the results indicated strong relationships among these key genes (Figure 3L).

### 3.4 PSO Skin Cell Composition and Cell Type Molecular Markers

**FIGURE 4:**
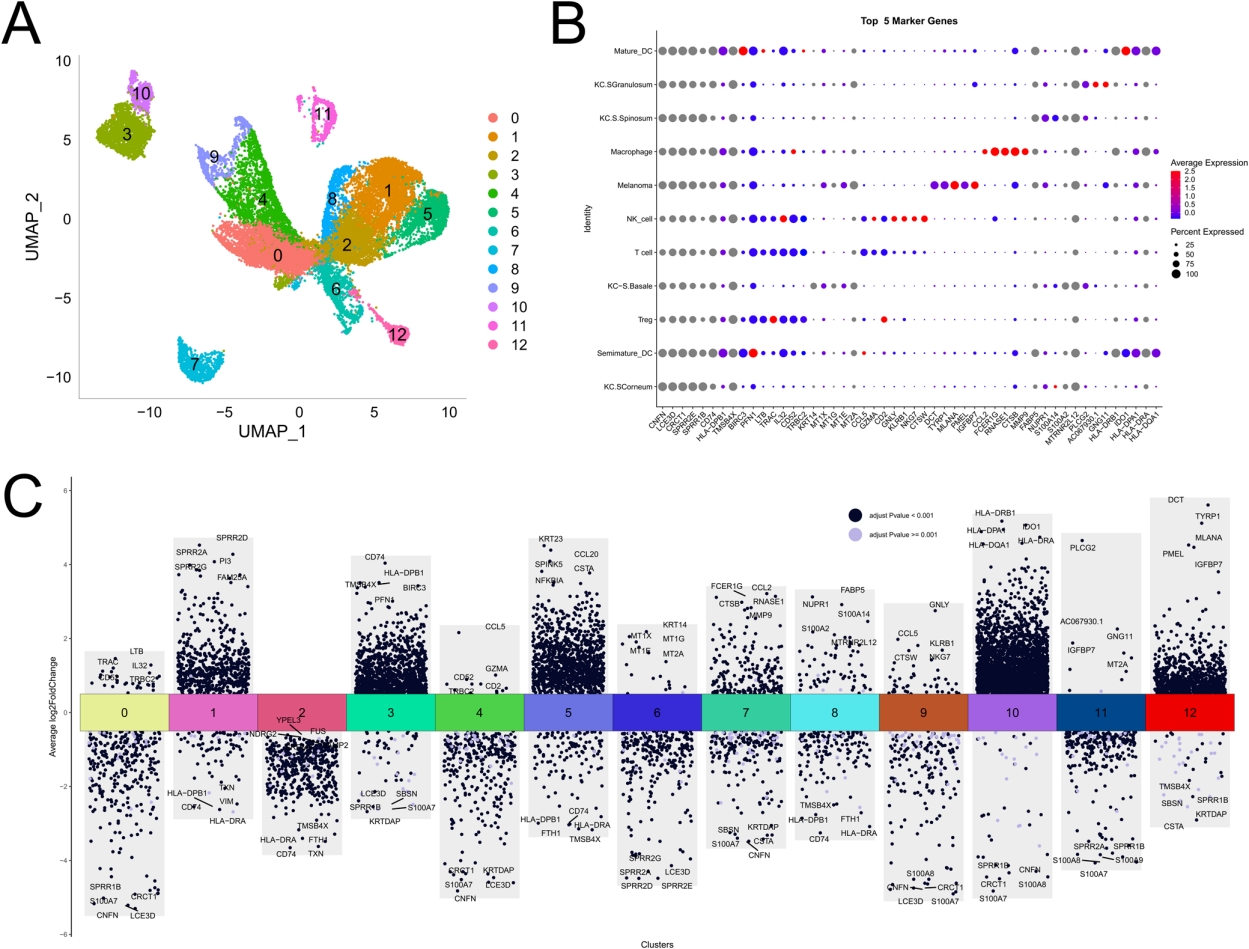
Skin single cell population and gene expression map in PSO. (A) UMAP diagram shows 13 cell clusters labelled with different colours. (B) The bubble map shows the expression of the top 5 marker genes in different cell clusters. (C) Volcano map showing the top 5 up‐regulated and down‐regulated genes of 13 subclusters.

To further explore the cellular composition of PSO skin. In this study, after quality control and standardization of scRNA‐seq data from the integrated PSO test set (13 psoriatic lesions and 5 normal skin tissues, the data are from GSE15177 in the GEO database), cells were subdivided into 13 cell clusters based on specific marker expression patterns (Figure 4A). They are Mature_DC, KC.SGranulosum, KC.S.Spinosum, Macrophage, Melanocytes, NK_cell, T cell, kc‐s.Besale, Treg, Semimature_DC, KC.SCorneum. Each cell cluster has its unique pattern of gene expression (Figure 4B). To compare the molecular markers in psoriatic and normal tissues, we performed differential expression analyses on all single‐cell sequencing data to identify the molecular markers for each cell type. The results showed that the volcano map showed differentially highly expressed genes in 13 cell clusters. CD74, HLA‐DPB1, TMSB4X, BIRC3 and PFN1 were highly expressed in Mature_DC cells. PLCG2, AC067930.1, BNG11, IGFBP7 and MT2 were highly expressed in KC.SGranulosum cells. SPRP2A, SPRP2G, SPRP2D, PI3 and FAM25A were highly expressed in KC.S.Spinosum. FCER1G, CCL2, CTSB, RNASE1 and MMP9 were highly expressed in Macrophage cells. DCT, TYRP1, MLANA, PMEL and IGFBP7 were highly expressed in melanocytes. GNLY, CCL5, KLRB1, CTSW and NKG were highly expressed in NK cells. CCL5, GZMA, CD52, TRBC2 and CD2 were highly expressed in T cells. MT1X, MT1E, KRT14, MT1G and MT2A were highly expressed in KC‐S.Basale. LTB, TRAC, IL32, CD52 and TRBC2 were highly expressed in Treg. HLA‐DRB1, HLA‐DPA1, HLA‐DQA1, IDO1 and HLA‐DRA were highly expressed in Semimature_DC. NUPR1, FABP5, S100A14, S100A2 and MTRNR2L12 were highly expressed in KC.SCorneum cells. These DEGs can be used as molecular markers for cell types (Figure 4C).

### 3.5 COX7C Significantly Correlated and Enriched in PSO Skin Cells

**FIGURE 5:**
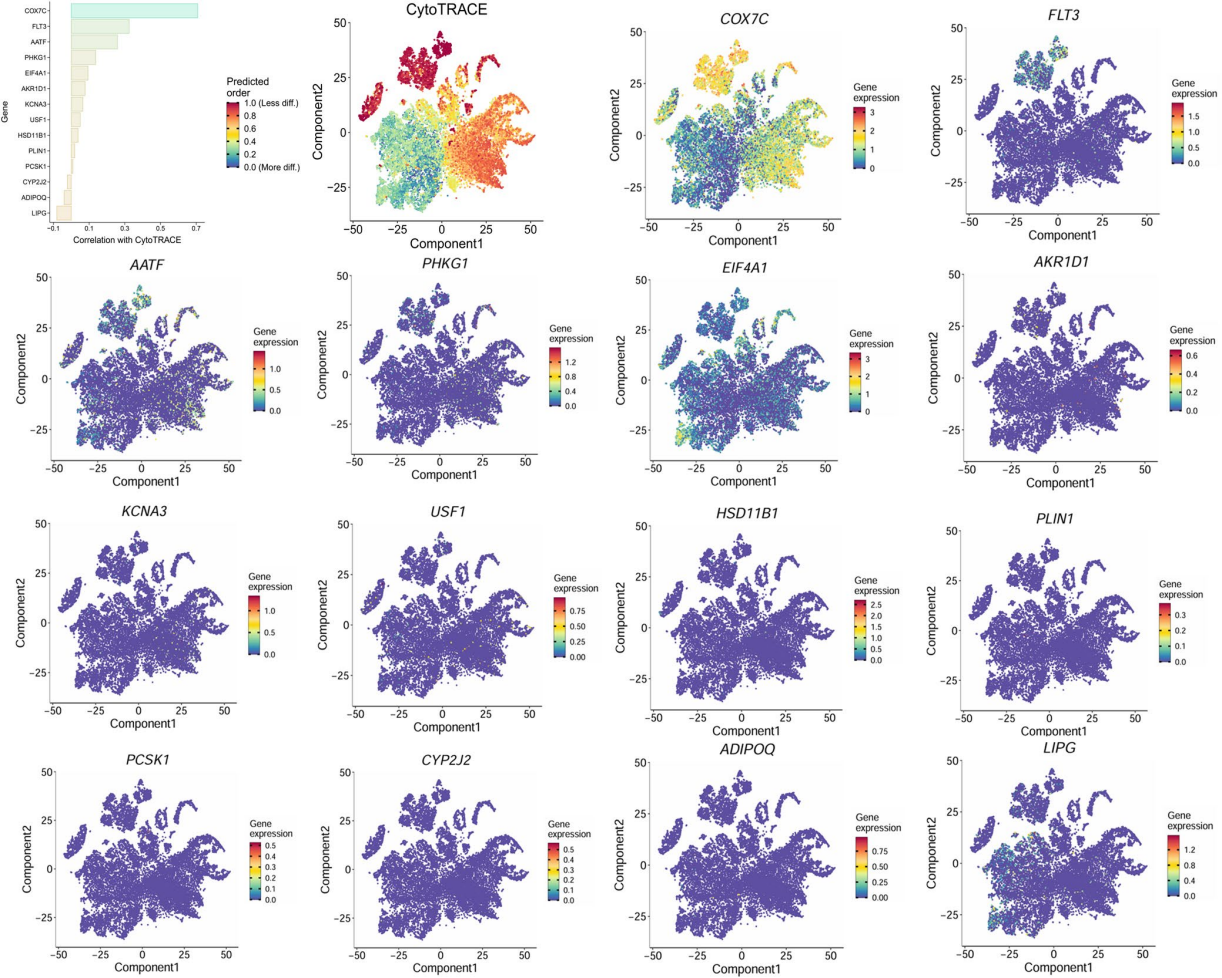
Characteristics of skin cell composition in PSO. (A) 14 PSO‐OB genes with the greatest influence on CytoTRACE prediction sequencing. (B) The t‐SNE diagram showed the CytoTRACE score distribution of 13 cell clusters, and the colours represented the predicted ranking scores.(C–P) Expression of 14 key genes in CytoTRACE score distribution in PSO cells.

To explore the developmental stages of each cell cluster in psoriatic skin, we used CytoTRACE for cell development trace analysis. The 14 PSO and OB genes most associated with CytoTRACE are COX7C, FLT3, AATF, PHKG1, EIF4A1, AKR1D1, KCNA3, USF1, HSD11B1, PLIN1, PCSK1, CYP2J2, ADIPOQ and LIPG (Figure 5A). CytoTRACE score was mapped to the profile (Figure 5B) to observe the distribution and expression of 14 PSO‐OB genes, and COX7C was the most relevant factor and enriched (Figure 5C–P).

### 3.6 24 TFs That Modulate Skin Cell Heterogeneity in PSO

**FIGURE 6:**
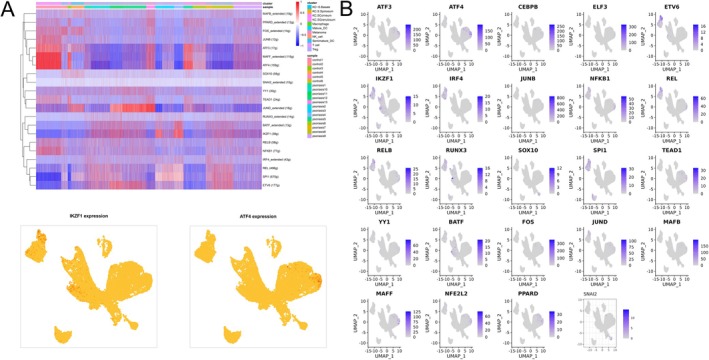
TF activity in PSO skin cell subtypes. (A) Thermogram showed the differences in TF activity between different cell subpopulations. (B) UMAP shows a normalized expression of TFs in cell subtypes.

For single‐cell transcriptome, the detection of TF activity can provide insight into PSO skin cell heterogeneity and analyse key regulatory mechanisms. We analysed the Top24 TFs that had the greatest effect on each single subtype of PSO and illustrated the intensity of regulation of each TF on different cell types (Figure 6A). We also show the corresponding TFs expression (Figure 6B).

### 3.7 PSO Skin Cell Subtypes Functionally Enriched in Lipid Metabolism

**FIGURE 7:**
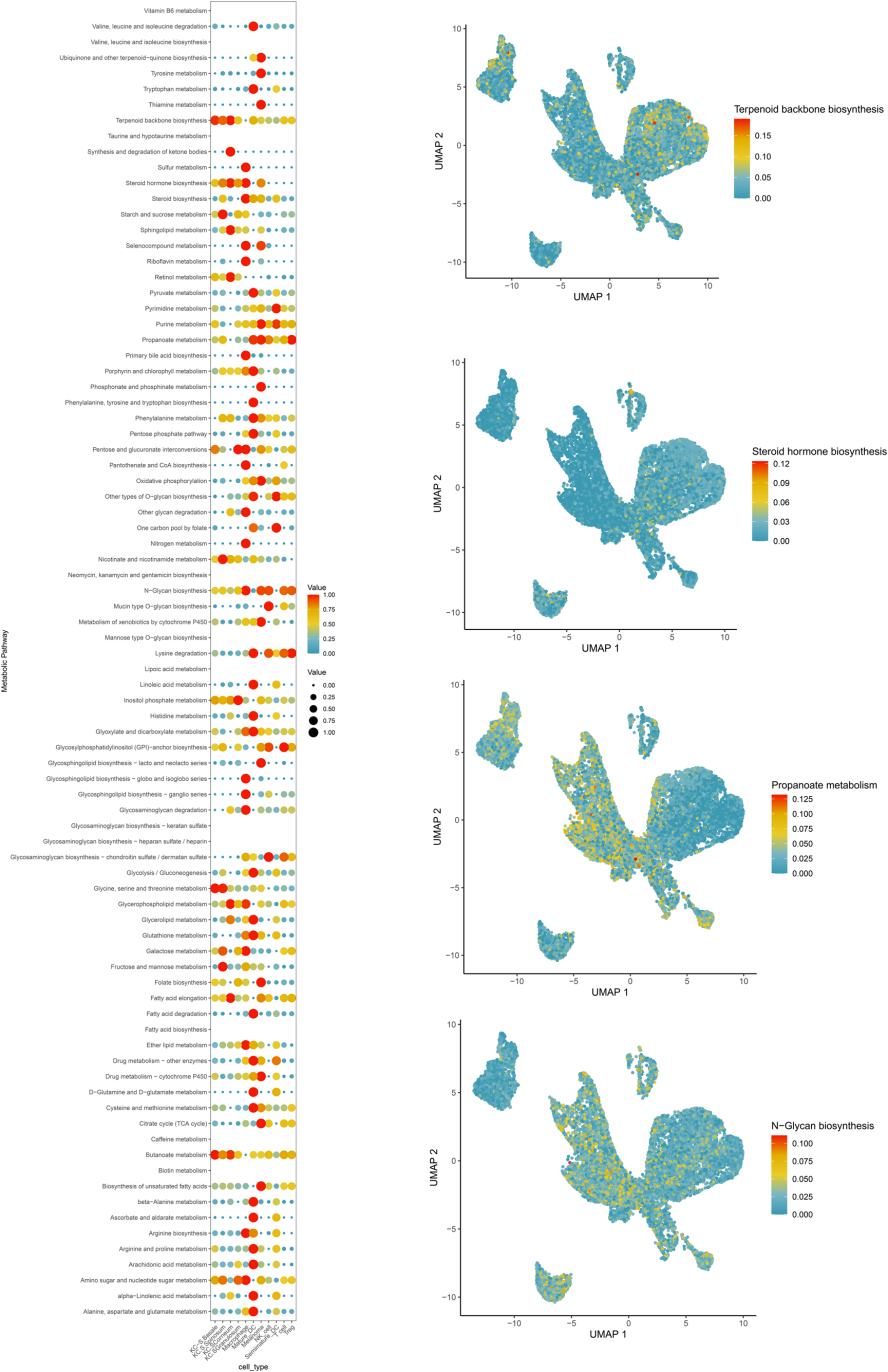
Functional characteristics of single‐cell subtypes. (A) The violin diagram showed differences in lipid metabolism between single‐cell subtypes. (B) UMAP plot coloured according to cluster for the lipid metabolism. The size of each dot represents the abundance of gene expression in the corresponding metabolic pathway of the cell type, and the intensity of the colour indicates the level of metabolic activity.

Subsequently, we evaluated the functional characteristics of psoriatic skin cell subtypes from a metabolic perspective. KC, macrophages, and mature DC are involved in Terpenoid backbone biosynthesis, Steroid hormone biosynthesis, Propanoate metabolism, and N‐glycan lipid metabolism, which is rich in biosynthesis (Figure 7A,B). These findings suggest that KC, macrophages, and mature DC exhibit specific functional diversity during lipid metabolism.

**FIGURE 8:**
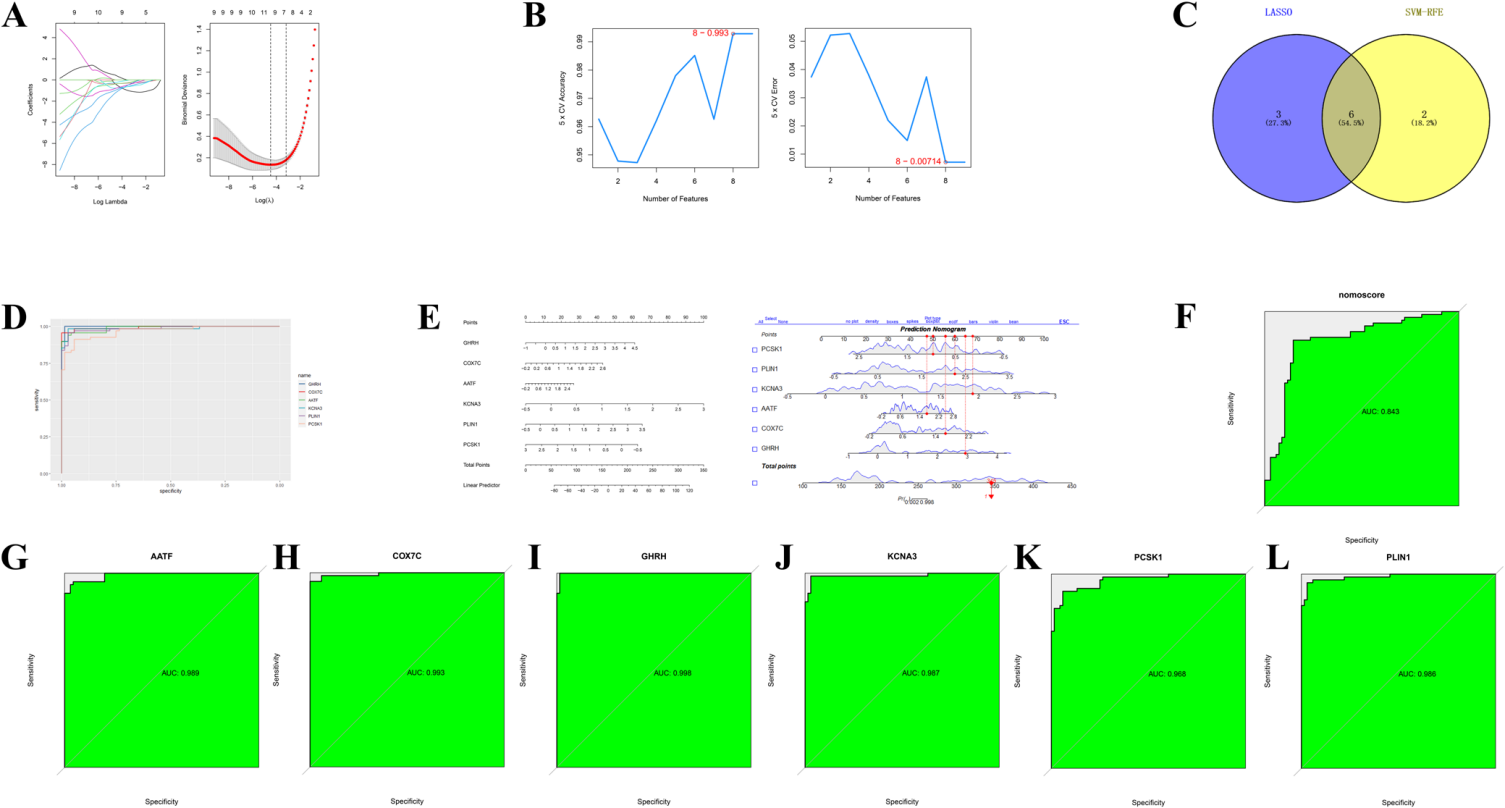
Screening and validation of PSO‐OB co‐diagnostic biomarkers. (A) The LASSO model shows the optimal coefficients and minimum lambda for the key genes of PSO‐OB. (B) The SVM‐RFE algorithm showed the selection of key gene features for PSO‐OB. (C) The Venn diagram shows the intersection of genes between LASSO and SVM‐RFE algorithms. (D) Diagnostic performance of the PSO‐OB hub gene was estimated by the ROC curve. (E) A nomogram of diagnostic biomarkers. (F) Calibration curves for predictive nomogram models. (G–L) ROC curve of genetic diagnosis effect of PSO‐OB hub.

Six diagnostic characteristic biomarkers of PSO‐OB. For the 19 key DEGs (EIF4A1, CYP2J2, AKR1D1, GHRH, HSD11B1, LIPF, COX7C, LIPG, FLT3, AATF, PHKG1, KCNA3, ADIPOQ, PLIN1, MCHR2, FGF8, PCSK1, USF1 and PRKACG) of PSO‐OB, the LASSO regression algorithm and SVM‐RFE algorithm were respectively employed to screen out potential common biomarkers that might be related to PSO‐OB. The LASSO regression algorithm identified nine variables (EIF4A1, CYP2J2, GHRH, HSD11B1, COX7C, AATF, KCNA3, PLIN1 and PCSK1) as the common diagnostic biomarkers for PSO‐OB (Figure 8A). The SVM‐RFE algorithm was used to identify subsets of 8 features (COX7C, FGF8, PLIN1, USF1, PCSK1, AATF, GHRH, KCNA3 in 19 genes; Figure 8B), and six overlapping features (AATF, COX7C, GHRH, KCNA3, PCSK1 and PLIN1) between the two algorithms were finally selected (Figure 8C). To explore the value of shared diagnostic genes in clinical applications, we used a logistic regression algorithm to establish diagnostic models for six genes and used the ROC curve model to judge the diagnostic effectiveness of genes. The ROC curve showed that the AUC value of the diagnostic model was 0.843, highlighting the high diagnostic efficiency of the model (Figure 8D–F). AUC values of AATF, COX7C, GHRH, KCNA3, PCSK1 and PLIN1 all exceeded 0.95, showing good diagnostic value (Figure 8G–L).

### 3.8 The Biomarkers Were Well Aligned With Psoriatic Unsaturated Fatty Acids

**FIGURE 9:**
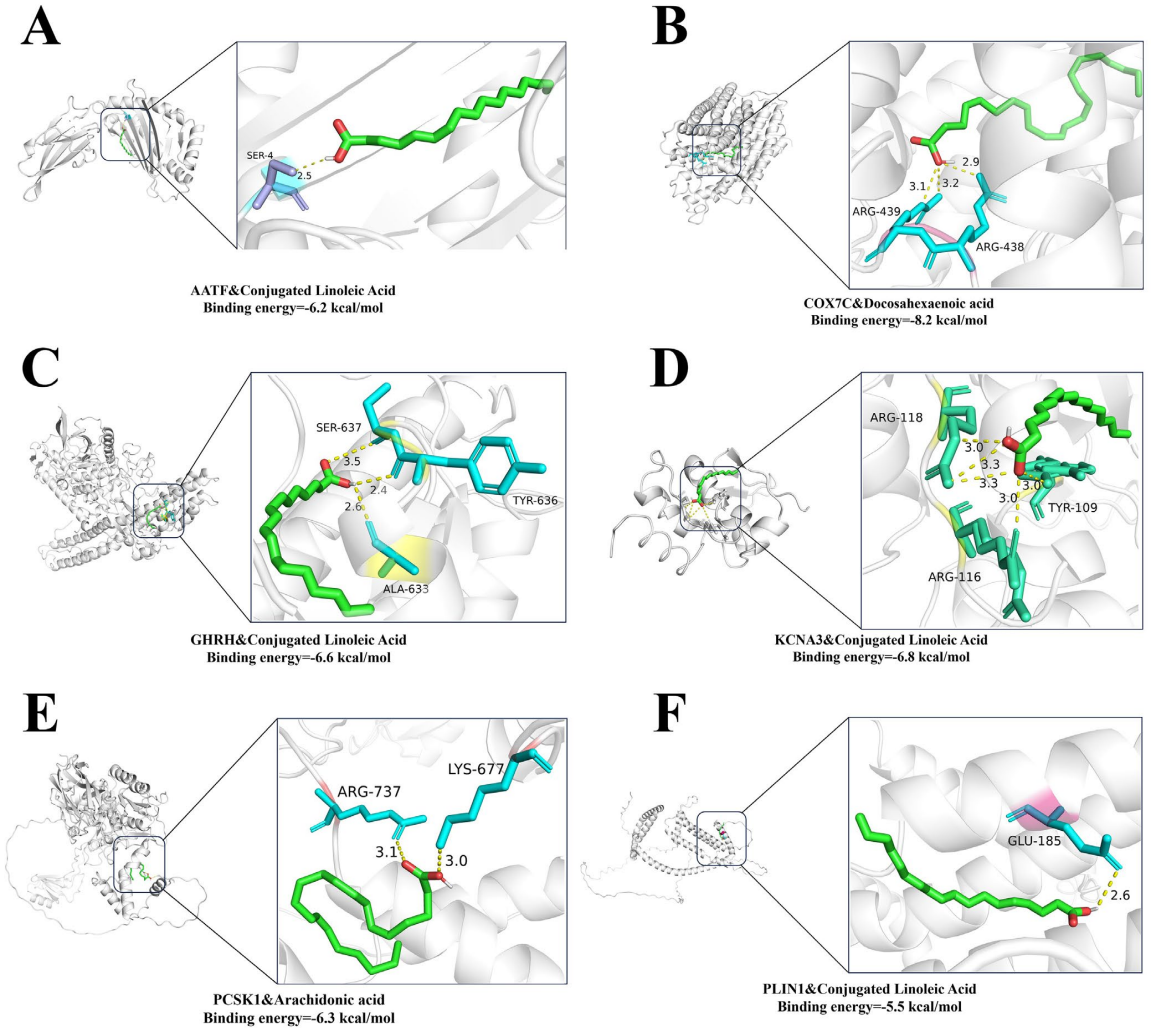
Six biomarkers were associated with the highest binding capacity of unsaturated fatty acids. (A–F) Molecular docking results of AATF, COX7C, GHRH, KCNA3, PCSK1 and PLIN1 with PSO closely related unsaturated fatty acids with the highest affinity. ALA, alanine residues; ARG, arginine residues; GLU, glutamic acid residue; LYS, lysine residues; SER, amino acid residues; TYR, tyrosine residue. Binding energy (kcal/mol), < −4.2 kcal/mol good binding activity, < −7 kcal/mol strong binding activity.

To further evaluate the binding of unsaturated fatty acids closely related to PSO with biomarkers, molecular docking was performed by autodocktools. The results showed that AATF interacted well with Conjugated Linoleic Acid (−6.2 kcal/mol), COX7C with Docosahexaenoic acid (−8.2 kcal/mol) and Conjugated Linoleic Acid (−7.4 kcal/mol), Eicosapentaenoic acid (−7.3 kcal/mol), Alpha‐Linolenic acid (−7.2 kcal/mol), Arachidonic acid (−7.2 kcal/mol), and it is compatible with Stearidonic acid (−7.1 kcal/mol). GHRH (−6.6 kcal/mol), KCNA3 (−6.8 kcal/mol), PCSK1 (−6.4 kcal/mol) and PLIN1 (−5.5 kcal/mol) interacted well with Conjugated Linoleic Acid (Figure 9).

### 3.9 COX7C Is Significantly Upregulated and Promotes Proliferation in Psoriatic Cells

**FIGURE 10:**
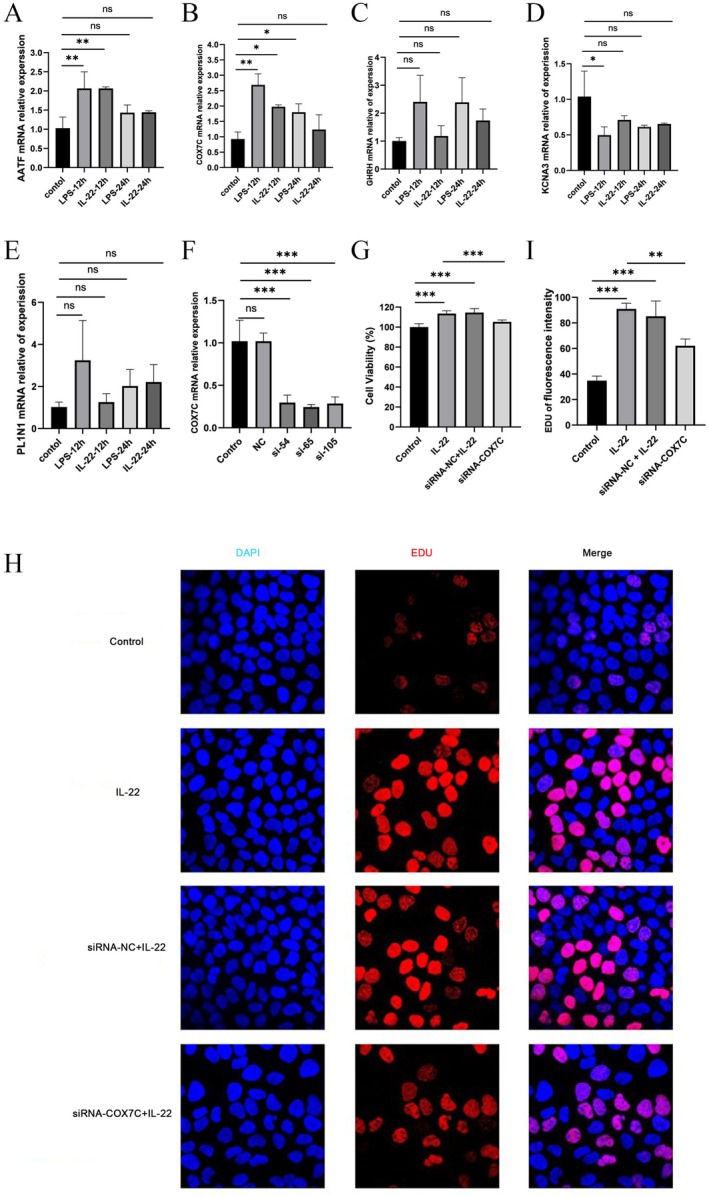
The mRNA expression levels of significantly expressed biomarker genes and their effects on the proliferation of PSO cells. (A) The mRNA level of the COX7C gene was significantly upregulated in HaCaT cells. (B–E) No statistically significant differences were observed on AATF, GHRH, KCNA3, PCSK1 and PLIN1. (F) Three targeted siRNAs were used for efficiency detection, and si‐65 had the highest inhibitory efficiency (≥ 50%). (G) After knockdown of COX7C, the viability of HaCaT cells was significantly reduced. (H, I) After knockdown of COX7C, the proliferation of HaCaT cells was inhibited. *p < 0.05, **p < 0.01, ***p < 0.001, ns = not significant.

A HaCaTcells exhibiting PSO‐like characteristics was established to further screen and verify potential biomarkers. After stimulation with IL‐22 or LPS for 12 h, the mRNA level of COX7C in HaCaT cells was significantly upregulated compared with the 0 h control group (*p* < 0.05/*p* < 0.01, statistically significant). Notably, 12 h was identified as the key time point for COX7C upregulation, providing a time gradient basis for selecting 12 h for subsequent functional verification. No significant statistical differences were observed in the mRNA levels of AATF, GHRH, KCNA3, PCSK1 and PLIN1 (Figure 10A–E). For GHRH, most samples failed to detect the gene in the qRT‐PCR assay, and the cycle threshold (*C*_t_) values of the few detectable samples were greater than 33, indicating that the expression level of GHRH in HaCaT cells was extremely low or undetectable.

To investigate the role of COX7C in the proliferation of HaCaT cells exhibiting PSO‐like characteristics, the overexpressed COX7C was knocked down using siRNA, and the knockdown efficiency was confirmed (Figure 10F). The CCK‐8 assay showed that IL‐22 significantly induced HaCaT cell proliferation (*p* < 0.001), whereas cell viability was markedly decreased after COX7C knockdown (*p* < 0.001); siRNA‐NC had no obvious interference effect. These results indicated that COX7C positively regulates the abnormal proliferation of psoriatic cells (Figure 10G).

Consistently, the EdU assay showed a significant increase in the number of EdU‐positive cells (reflected by red fluorescence intensity) in the IL‐22 group, confirming that IL‐22 successfully induced abnormal proliferation of HaCaT cells (*p* < 0.001). No significant difference in the number of EdU‐positive cells was observed between the siRNA‐NC + IL‐22 group and the IL‐22 group, excluding the interference of the transfection operation on cell proliferation. After COX7C knockdown, the number of EdU‐positive cells was significantly reduced (Figure 10H), and COX7C knockdown could inhibit IL‐22‐induced cell proliferation (*p* < 0.01) (Figure 10I). The results of the EdU assay were consistent with those of the CCK‐8 assay, confirming the reliability of the conclusion that COX7C knockdown inhibits the proliferation of psoriatic cells.

### 3.10 COX7C Positively Regulates the Inflammatory Response in PSO

**FIGURE 11:**
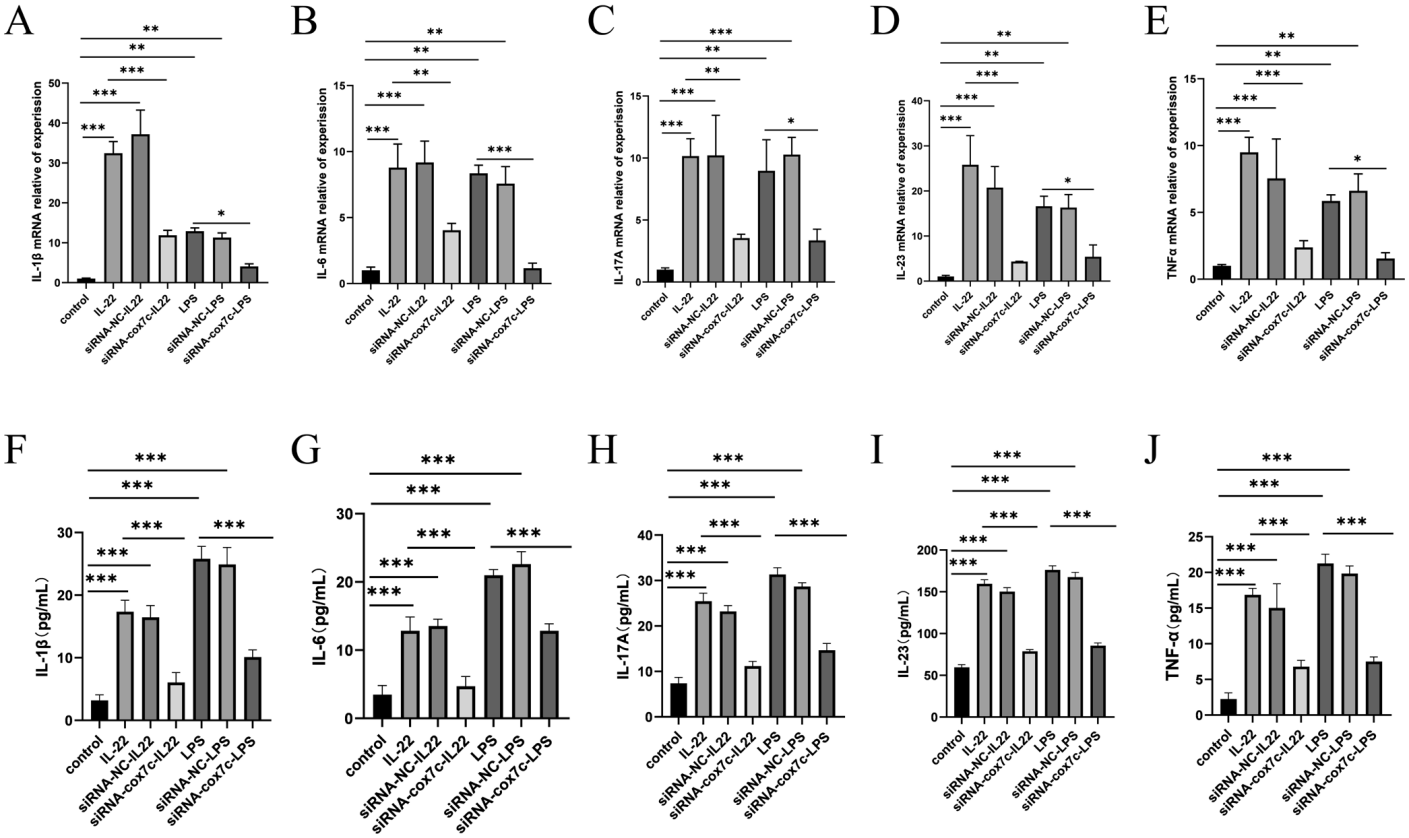
The mRNA expression levels and protein concentrations of five core inflammatory factors in HaCaT cells exhibiting PSO‐like characteristics after COX7C knockdown. (A–E) the mRNA expression levels of pro‐inflammatory factors IL‐1β, IL‐6, IL‐23, IL‐17A and TNF‐α were significantly decreased following COX7C knockdown, with statistically significant differences. (F–J) the detection concentrations of inflammatory factors IL‐1β, IL‐6, IL‐23, IL‐17A and TNF‐α in cell supernatants were significantly reduced after COX7C knockdown, and the differences were statistically significant. *p < 0.05, **p < 0.01, ***p < 0.001.

By silencing the COX7C gene using siRNA, the effect of this gene on the inflammatory response in the HaCaT cells exhibiting PSO‐like characteristics was investigated, and its role in the pathogenesis of PSO was clarified. The results of qRT‐PCR showed that in the IL‐22 and LPS models, the mRNA levels of pro‐inflammatory factors IL‐1β (*p* < 0.001 and *p* < 0.01), IL‐6 (*p* < 0.001 and *p* < 0.01), IL‐23 (*p* < 0.001 and *p* < 0.01), IL‐17A (*p* < 0.001 and *p* < 0.01) and TNF‐α (*p* < 0.001 and *p* < 0.01) were significantly upregulated. After silencing COX7C, the mRNA expressions of pro‐inflammatory factors IL‐1β (*p* < 0.001 and *p* < 0.05), IL‐6 (*p* < 0.01 and *p* < 0.001), IL‐23 (*p* < 0.001 and *p* < 0.05), IL‐17A (*p* < 0.01 and *p* < 0.05) and TNF‐α (*p* < 0.001 and *p* < 0.05) were significantly decreased. si‐NC had no interference, proving that COX7C targets and regulates the PSO inflammatory pathway (Figure 11A–E). ELISA detection of the protein concentration of inflammatory factors in cell supernatants showed that the protein concentrations of inflammatory factors (IL‐1β, IL‐6, IL‐23, IL‐17A and TNF‐α) were significantly increased in both IL‐22 and LPS stimulation groups (all *p* < 0.001 for both models). After silencing COX7C, the protein concentrations of inflammatory factors IL‐1β (*p* < 0.001 and *p* < 0.001), IL‐6 (*p* < 0.001 and *p* < 0.001), IL‐23 (*p* < 0.001 and *p* < 0.001), IL‐17A (*p* < 0.001 and *p* < 0.001) and TNF‐α (*p* < 0.001 and *p* < 0.001) were significantly decreased (Figure 11F–J). The combined verification of qPCR and ELISA confirmed that the transcription and protein levels of the five core inflammatory factors of PSO were consistent, proving that COX7C positively regulates the PSO inflammatory response.

## 4 Discussion

PSO is a chronic, immune‐mediated inflammatory skin disease characterized by abnormal proliferation of keratinocytes and persistent skin inflammation. Its pathogenesis is complex and has not been fully elucidated. Increasing clinical epidemiological evidence indicates that OB and PSO have a close comorbidity relationship. The probability of OB patients developing PSO is significantly higher, and the prognosis is also worse. PSO is characterized by the proliferation of Th1, Th17 and Th22 cells, resulting in the production of pro‐inflammatory mediators interferon‐gamma, TNF‐α, IL‐6 and IL‐2 [15]. OB may induce Th17 cells to secrete IL‐17, and white adipose tissue is a key site for the formation of pro‐inflammatory adipokines such as IL‐6, TNF‐α, leptin and resistin. Studies have shown that TNF‐α and IL‐6 secreted by adipose tissue may contribute to the inflammatory state of PSO and that both leptin and resistin levels are high in obese patients with PSO, and their plasma concentrations correlate with the severity of PSO [7]. Multiple studies have shown TNF‐α, IL‐6, Krt16, IL‐21 and nestin as biomarkers for the diagnosis of PSO in obese patients, revealing the bidirectional relationship between PSO and OB [16, 17]. However, most of the existing studies lack systematic exploration of the molecular bridge mechanisms and core regulatory biomarkers that connect the occurrence and progression of OB and PSO. Therefore, the main scientific objective of this study is to clarify the causal relationship between PSO and OB, determine the key hub genes and key molecular pathways mediating the interaction between PSO and OB, and further explore the key functional genes and potential therapeutic targets behind the metabolic‐inflammation imbalance of PSO.

In this study, integrative bioinformatics analysis and functional validation led to innovative key findings. First of all, the MR Analysis using a large genome‐wide association dataset statistically confirmed that OB is an independent high‐risk factor for PSO, thereby strengthening the epidemiological link between OB and PSO. Second, through the intersection of PSO DEGs and WGCNA module genes with OB‐related genes, 19 specific key genes of PSO‐OB were screened out, and AUC verification verified the excellent stability and diagnostic specificity of these genes, providing a concentrated and reliable gene cluster for exploring the OB‐PSO interaction mechanism. The functional enrichment analysis conducted through GSVA and GSEA further demonstrated that these genes were significantly related to lipid metabolism pathway, thermogenesis, and Ras signalling pathway. This confirms the metabolic link between PSO and OB.

Innate immunity and adaptive immunity are involved in the pathogenesis of PSO. KC and immune cells are significantly increased in PSO lesions. During the pathogenesis of PSO, KC is the first response cell that, when activated, secretes innate immune mediators leading to inflammation. Immune cells interfere with the metabolism of KC, resulting in increased activation. Given the pathogenesis of KC and immune cells in PSO. Thirdly, we explored the relationship between PSO and OB co‐key DEGs and psoriatic skin cells. Single‐cell transcriptome analysis of psoriatic data showed that psoriatic skin cells were mainly composed of KC, DC, macrophages, NK cells, T cells, Treg cells and melanocytes. Molecular markers in each cell subtype were identified. Further exploring the developmental stages of each skin cell subtype, COX7C was found to be the most relevant factor and significantly enriched.

scRNA‐seq describes molecular heterogeneity at the single‐cell level [18, 19]. The heterogeneity of psoriatic skin cells is mainly reflected in the functional differences between different cell types and the different states within the same cell type. This heterogeneity not only exists among epidermal cells but also involves the diversity and dynamic regulation of dermal cells and immune cells [20, 21]. The difference in the activity of TFs is one of the important factors leading to this heterogeneity. In PSO, some TFs such as STAT3 and IL‐17A show abnormal activity. The abnormal activity of these TFs not only affects the normal physiological function of skin cells, but also promotes inflammatory response, excessive proliferation and differentiation of KC, and regulates the activation of immune cells [22]. In the epidermal and immune cell subsets of PSO, Top24 TFs with the greatest influence on PSO cell subsets were identified. Including ATF3, ATF4, CEBPB, ELF3, EVT6, IKZF1, IRF4, JUNB, NFKB1, REL, RELB, RUNX3, SOX10, SPI1, TEAD1, YY1, BATF, FOS, JUND, MAFB, MAFF, NFE2L2, PPA RD and SNA12. Notably, ATF4 is highly regulatory in KC and T cell subsets. ATF4 gene is a widely expressed stress response gene in cells, involved in a variety of stress responses, such as hypoxia, and malnutrition. ATF4, as a TF, can regulate cells to adapt to various stressful environments. CD4 T cell function depends on catabolic glycolysis and glutamine catabolism mediated by ATF4, which regulates CD4+ T cell‐mediated immune response by driving metabolism [23]. Studies have shown that patients with PSO develop a strong neuroendocrine response in the presence of acute stressors, increasing vulnerability to psoriatic activity [24, 25]. In breast cancer and melanoma studies, ATF4 is highly expressed in a variety of tumours and supports tumour growth and progression by promoting tumour‐associated fibroblast function [26, 27]. Inhibition of ATF4 can significantly inhibit tumour growth and metastasis, suggesting that ATF4 may be a potential target for cancer therapy.

PSO and metabolic syndrome involve imbalances in inflammatory response and immune regulation. Metabolic syndrome increases the risk of PSO, and analysis of metabolic factors suggests that OB is a central factor in this association [28]. Obese individuals with BMI ≥ 30 significantly increase the incidence of PSO, leading to worsening of the condition and poor response to treatment [29, 30]. The symptoms of PSO may also affect a patient's metabolic status. The study found that abnormal metabolism of 25‐(OH) D3, Cu/Zn and HDL‐C may be involved in the pathogenesis of PSO by improving the inflammatory response, producing reactive oxygen species, or eliminating immune system inhibition [31, 32]. Lipid disturbance in PSO involves not only systemic dyslipidaemia but also changes in lipid metabolites in the skin tissue. Total lipids, phospholipids, triacylglycerol and cholesterol are increased in the blood and epidermis of PSO, and the increased levels of free cholesterol and phospholipids in the epidermis are proportional to the severity of PSO [33]. This mutual influence can lead to worsening or recurrent episodes in patients, which can make treatment more difficult.

We evaluated the functional characteristics of psoriatic skin cell subtypes from a metabolic perspective. It was found that KC, macrophages, and mature DC showed specific functional diversity during lipid metabolism, especially mature DC. Key metabolic pathways such as glycolysis, tricarboxylic acid cycle, lipid metabolism, and amino acid metabolism are essential for the regulation of KC and immune cells [34]. In an obese state, the function of macrophages and lymphocytes in adipose tissue changes, leading to immune system disorders [35]. Psoriatic DC overmature, increased phagocytosis, and excessive secretion of IL‐23 [36]. DC has high levels of neutral lipids, including triglycerides, diglycerides, phosphatidyl ethanol and phosphatidylcholine. Transcriptional analysis showed that disease‐associated DC in imiquimod mice was involved in the expression of multiple genes involved in lipid metabolism and autophagy, and the associated immune function was correspondingly dysregulated [37]. Psoriatic epidermal DC accumulates cytolipids, which are associated with immune activation and oversecretion of IL‐23. The immune response triggered by different lipids is affected by differences in DC subsets, unstable lipid types, and cytokine microenvironments. The epidermal DC presents the new lipid antigen to CD1A‐producing reactive IL‐17 T cell CD1a via CD1a [38]. It has been confirmed that the PSO skin cell subtypes possess unique functional advantages in lipid metabolism, and has revealed the cellular heterogeneity basis of metabolic disorders in PSO lesions.

Fourthly, by integrating the LASSO regression and SVM‐RFE algorithms, 6 overlapping feature‐sharing genes (AATF, COX7C, GHRH, KCNA3, PCSK1, PLIN1) were selected. In addition, the value of diagnostic genes in clinical applications was shared by the logistic regression algorithm and ROC analysis. COX7C is a subunit of cytochrome c oxidase that transfers electrons from cytochrome c to oxygen. Studies have shown that the relative expression level of COX7C is increased in diabetes‐related sepsis and cutaneous squamous cell carcinoma [39, 40]. In chronic kidney disease, COX7C is specifically down‐regulated [41]. Cox7c is widely expressed in the mitochondria of major vascular endothelial cells. Ischemia/reperfusion injury results in decreased Cox7c expression, whereas DL‐3‐n‐Butylphthalide (NBP) increases the expression of Cox7c to maintain the integrity of the blood–brain barrier [42]. AATF is an anti‐apoptotic TF gene, which can mediate its anti‐apoptotic effect by interfering with MAP3K12, regulating AKT1 to induce or inhibit p53‐mediated apoptosis [43, 44, 45]. AATF is overexpressed in human head and neck squamous cell carcinoma and positively regulates cell proliferation and colony formation. AATF may promote cisplatin resistance and reduce apoptosis by regulating STAT3/survivin signalling and may be a potential therapeutic target for head and neck squamous cell carcinoma [46].

OB and high‐fat diets increase the concentration of fatty acids, and dietary fatty acids appear to be key amplifiers of psoriatic inflammation. Studying the regulatory mechanisms of fatty acids in PSO may help improve PSO treatment response and comorbidities [32]. The study evaluated the binding capacity of six common biomarkers for diagnosing PSO‐OB and 10 unsaturated fatty acids closely related to PSO through molecular docking. It was found that AATF, COX7C, GHRH, KCNA3, PCSK1 and PLIN1 had good binding activity with Conjugated Linoleic Acid, and COX7C was the strongest. It has strong binding activity with Docosahexaenoic acid, Eicosapentaenoic acid, Alpha‐Linolenic acid, Arachidonic acid and Stearidonic acid. Linoleic acid is an essential fatty acid that plays an important role in lowering cholesterol and repairing disorders. Compared with healthy controls, plasma linoleic acid levels in PSO patients were significantly lower [47]. Linoleic acid may inhibit the proinflammatory function of PSO and PSA, and protect the immunosuppression of Tregs in PSA. In PSO, linoleic acid metabolism is negatively correlated with AMPK and PI3‐Akt signalling pathways [48]. In a mouse breast tumour model, linoleic acid promotes initial T cell apoptosis and inhibits TNF‐α production [49]. Ingestion of linoleic acid may have therapeutic effects in cellular autoimmune diseases characterized by type 1 immune responses characterized by excessive production of IFN‐γ and TNF‐α. Identification and analysis of linoleic acid metabolism to understand the pathogenesis of PSO is expected to provide a promising candidate for adjuvant therapy of PSO disease. It was initially confirmed that these six biomarkers have a high binding affinity with the unsaturated fatty acids associated with PSO, and the reliable molecular interaction between the core biomarkers and the abnormal lipid metabolism in psoriatic oedema was verified.

Finally, through experiments using an in vitro cell model (HaCaT cells exhibiting PSO‐like characteristics) and dual verification by qPCR and ELISA, it was demonstrated that COX7C is significantly upregulated in PSO‐like keratinocytes, capable of promoting the proliferation of keratinocytes, and positively regulating the inflammatory response of PSO by synchronously regulating the transcription and protein expression levels of five core inflammatory factors (IL‐1β, IL‐6, IL‐23, IL‐17A and TNF‐α) in PSO. However, the HaCaT cells exhibiting PSO‐like characteristics used in the in vitro experiments are only employed to explore certain molecular phenotypes related to PSO, rather than being used as a complete in vitro model of PSO. Further in vivo verification is needed to confirm these results.

In addition to the above findings, there are some limitations to this study. First, the study population was limited to European ancestry, which limits the generalisation of our results to other populations. Second, we used the genome‐wide significance threshold *p* value < 5 × 10^−6^ instead of *p* < 5 × 10^−8^ to generate sufficient IV for MR Analysis. However, it is considered a suitable threshold and is used for MR Analysis. Third, despite our findings on the variability of PSO vulgaris in obese people, more studies are needed to uncover the underlying mechanisms. Last, all predictions need to be confirmed with laboratory data, and the expression of the aforementioned PSO‐OB biomarker genes needs to be validated using large‐scale samples in future studies. Further, future studies aim to confirm gene interactions, regulatory associations between TFs and genes, and possible metabolic pathways by which these genes are altered.

Overall, this study has demonstrated that the lipid metabolism disorder caused by OB is a key initiating factor in the pathogenesis of PSO. The 19 key genes of PSO‐OB and the six selected biomarkers (AATF, COX7C, GHRH, KCNA3, PCSK1 and PLIN1) serve as crucial molecular bridges connecting the abnormal metabolic disorders of OB and the inflammatory lesions of PSO. The abnormally expressed COX7C is a positive regulatory factor for keratinocyte proliferation and inflammatory response in PSO, and can serve as a potential diagnostic biomarker and therapeutic target for obese patients with PSO. Subsequent studies will focus on supplementing clinical sample verification and in vivo animal experiments to confirm the clinical diagnostic efficacy and therapeutic potential of the six biomarkers such as COX7C. In addition, further mechanism experiments will be conducted to explore the specific regulatory pathways of COX7C in regulating lipid metabolism and inflammatory response in PSO. This will provide a more comprehensive and precise theoretical basis for the clinical prevention and targeted treatment of OB concurrent PSO.

## Author Contributions

**Yongfeng Chen:** conceptualization, methodology, writing – review and editing, resources, project administration, supervision. **Shougang Liu:** conceptualization, methodology, data curation, software, investigation, validation, formal analysis, supervision, resources, visualization, writing – review and editing. **Ruxue Han:** conceptualization, methodology, investigation, data curation. **Fanghua Liu:** methodology, software, data curation, formal analysis, investigation, writing – original draft, validation. **Hang Su:** validation, data curation.

## Funding

The authors have nothing to report.

## Ethics Statement

The authors have nothing to report.

## Consent

The authors have nothing to report.

## Conflicts of Interest

The authors declare no conflicts of interest.

## Supporting information


**Figure S1:** Research design framework: (a) The MR Study was built on three main assumptions: (1) The genetic variation used as an instrumental variable in the analysis (IV) should be strongly associated with obesity; (2) genetic variants used for obesity IV should not be associated with known confounders; (3) genetic variants as IV should affect the risk of psoriasis related diseases only through obesity. (b) Bidirectional MR design.

**Table S1:** Basic information on OB and PSO‐related data for MR Analysis.

**Table S2:** Information of IVs for significant exposure‐outcome pairs in MR analyses.

**Table S3:** Sensitivity analysis of MR.

## Data Availability

The raw data supporting the conclusions of this manuscript are included in the article, further queries can be directed to the corresponding author.
