# Supplementary figures and images for: Psoriasis–Obesity‐Related Gene COX7C Promotes Keratinocyte Proliferation and Positively Regulates Inflammatory Responses in Psoriasis
Source: J Cell Mol Med. 2026 Jul 22;30(14):e71277. doi: 10.1111/jcmm.71277 (PMC13392194; doi:10.1111/jcmm.71277)

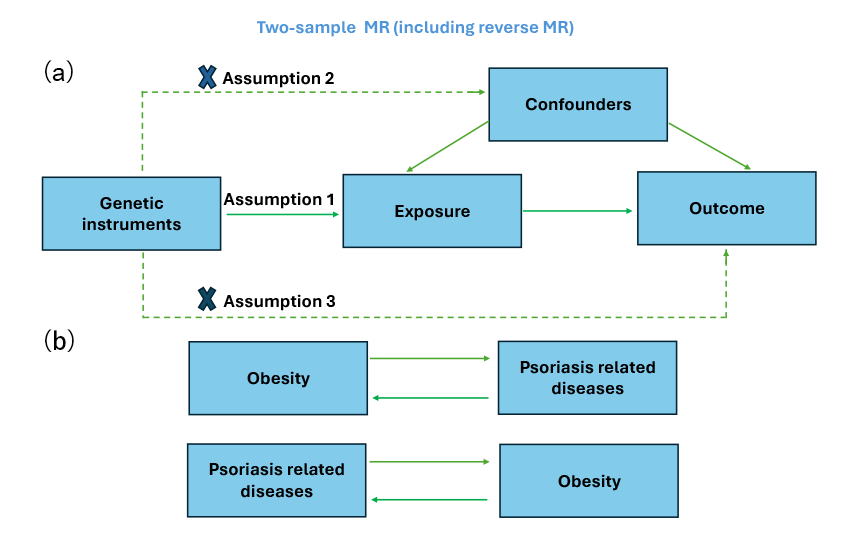

Supplement: Supplementary file 1 — Figure S1: Research design framework: (a) The MR Study was built on three main assumptions: (1) The genetic variation used as an instrumental variable in the analysis (IV) should be strongly associated with obesity; (2) genetic variants used for obesity IV should not be associated with known confounders; (3) genetic variants as IV should affect the risk of psoriasis related diseases only through obesity. (b) Bidirectional MR design. [file JCMM-30-e71277-s003.png]
